# Calibration of Acousto-Optic Interaction Geometry Based on the Analysis of AOTF Angular Performance

**DOI:** 10.3390/ma16103708

**Published:** 2023-05-13

**Authors:** Hao Zhang, Huijie Zhao, Qi Guo, Yan Xuan

**Affiliations:** 1 School of Instrumentation Science and Opto-Electronics Engineering, Beihang University, Beijing 100191, China; hao2017@buaa.edu.cn (H.Z.);; 2 Institute of Artificial Intelligence, Beihang University, Beijing 100191, China

**Keywords:** AOTF, acousto-optic interaction geometry, polar angular analysis, tolerance analysis

## Abstract

Acousto-optic interaction geometry determines the spectral and spatial response of an acousto-optic tunable filter (AOTF). The precise calibration of the acousto-optic interaction geometry of the device is a necessary process before designing and optimizing optical systems. In this paper, we develop a novel calibration method based on the polar angular performance of an AOTF. A commercial AOTF device with unknown geometry parameters was experimentally calibrated. The experimental results show high precision, in some cases falling within 0.01°. In addition, we analyzed the parameter sensitivity and Monte Carlo tolerance of the calibration method. The results of the parameter sensitivity analysis show that the principal refractive index has a large influence on the calibration results, while other factors have little influence. The results of the Monte Carlo tolerance analysis show that the probability of the results falling 0.1° using this method is greater than 99.7%. This work provides an accurate and easy-to-perform method for AOTF crystal calibration and can contribute to the characteristic analysis of AOTFs and the optical design of spectral imaging systems.

## 1. Introduction

An AOTF device is a spectral-splitting device based on the acousto-optic effect [1]. When compared with traditional optical splitters, such as prism, grating and interferometer, it has advantages in terms of a large-angle aperture, high spectral resolution, arbitrary wavelength configuration, fast tuning speed and it does not require moving elements [2,3]. Therefore, it is widely used in many applications, such as spectral imaging [4,5], polarization analysis [6,7], stereoscopic imaging [8] and notch filtering [9], etc. In addition, in the case of monochromatic input light, AOTFs also have the abilities of spatial filtering and edge enhancement [10,11]. There are two basic configurations, collinear and noncollinear, for AOTFs. Furthermore, in the collinear configuration, the interacting optical and acoustic waves propagate in identical directions, while the directions of the optical and acoustic waves are different in the noncollinear configuration. The first AOTF with a collinear design was reported by Harris and Wallace in 1969 [12]. Subsequently, Chang described noncollinear AOTFs in 1974 [13], which are commonly used today. Noncollinear AOTFs have some advantages, as a larger angular aperture and more materials with a large acousto-optic figure of merit can be chosen. Among them, TeO_2_ crystals are very suitable for noncollinear AOTFs that cover the spectral range of 350–4500 nm [14,15].

**Figure 1:**
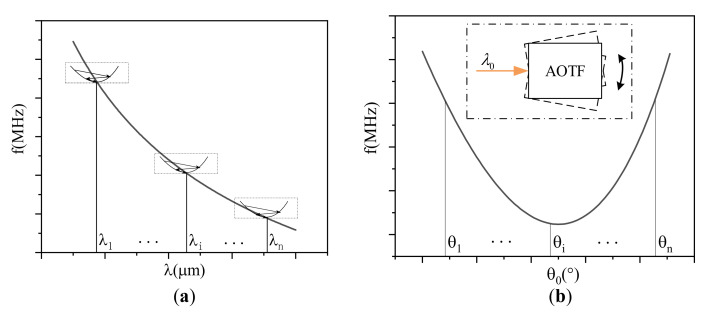
Comparison of operating principles. (**a**) Calibration method by the tested tuning curves under the parallel tangent condition. (**b**) Calibration method using polar angular analysis, which has no specific requirement of incident angles.

Acousto-optic interaction geometry is a key inherent property of an AOTF device, which greatly affects the spectral and spatial response of the device. Previously, Voloshinov analyzed the acousto-optic effect under three kinds of acoustic cut angles, and the results showed that acousto-optic interaction geometry affects the angular apertures and spectral resolution of an AOTF device [16]. In addition, acousto-optic interaction geometry will also affect the tuning relationship, sound field distribution, aberration and chromatic aberration characteristics of the device, thus greatly affecting all aspects of the AOTFs in characteristic analysis and optical system design [4,17]. Therefore, AOTFs must be designed in detail to meet the special output requirements. Chang first proposed the parallel tangent condition for the design of noncollinear AOTFs with large angular apertures in the 1970s [13]. Yano discussed some properties of AOTFs using the simplified treatment [18]. Moreover, Gass corrected the birefringence approximation for the accurate design of the acousto-optic interaction geometry in the analysis [19]. These theories are all typically under the parallel tangent condition. For the non-parallel tangent condition, Yushkov expressed an exact phase-matching calculation equation at an arbitrary incident angle and recently proposed an alternative method for analyzing the Bragg angle curve in wide-angle AOTFs [10,20]. Zhang discussed the function of the phase mismatching condition and proposed a new tuning method with a non-radio-frequency signal [21]. After the processes of analysis and design, an AOTF will be manufactured using a series of fabrication technologies. Many steps are involved in the fabrication of AOTFs, including X-ray orientation, cutting, polishing, transducer orientation and fabrication, mounting and grinding [22]. The fabrication technologies of an AOTF are so complex that they easily lead to machining tolerances between the designed and actual device. If the designed values were used for device characteristic analysis and optical system design, it would lead to inaccurate results. Therefore, it is vital to calibrate the acousto-optic interaction geometry of an actual AOTF before use. In the past, our team proposed an acousto-optic interaction geometry calibration method by the tested tuning frequency curve under the parallel tangent condition [23]. However, this multi-wavelength method is not conducive to calibration accuracy as it relies on more constant parameters. In addition, to find the parallel tangent condition, as shown in Figure 1a, the incident angles must be adjusted accurately, which requires high precision in the experiment.

To overcome these shortcomings, here we develop a calibration method based on the polar angular performance of an AOTF. Firstly, we establish an AOTF angular frequency relationship model that can be solved analytically. Moreover, based on this model, a novel method is developed to calibrate the acousto-optic interaction geometry of an actual AOTF device. It does not introduce principal refractive index errors between multiple wavelengths and works with a single monochromatic light source. Furthermore, this method does not depend on determining characteristic incident angles, as shown in Figure 1b. Finally, using the principle of the minimum root mean square error (RMSE) between the measured and theoretical data, the acousto-optic interaction geometry of the actual AOTF device can be calculated through the use of the parameter traversal method. This method is an improvement of the calibration process in terms of simplicity and robustness and has been tested with high precision in experiments. Simultaneously, the method analyzes the influence of crystal constants on calibration results in the visible range. This work is significant and provides a database for a range of research related to AOTF devices.

## 2. Methods

**Figure 2:**
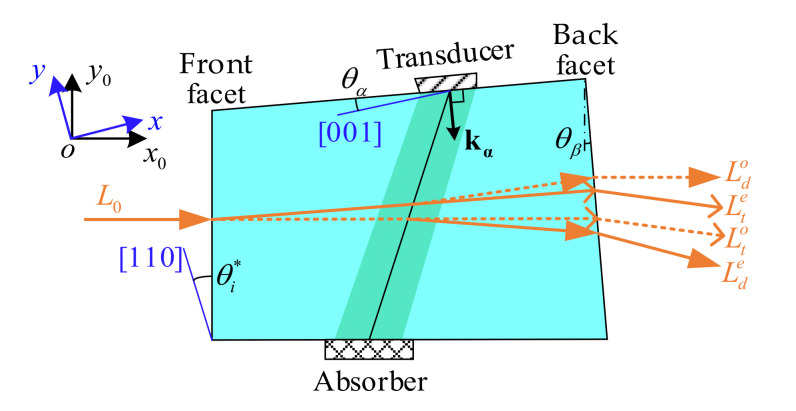
Acousto-optic interaction geometry of an AOTF. The $\theta_{i}^{*}$ is the front facet angle between the front facet and the crystal axis [110]. The $\theta_{\alpha }$ is the acoustic cut angle between the transducer surface and the crystal axis [001]. The $\theta_{\beta }$ is the back facet angle between the back and front facets.

The acousto-optic interaction geometry of an AOTF refers to the front facet angle ($\theta_{i}^{*}$), the acoustic cut angle ($\theta_{\alpha }$) and the back facet angle ($\theta_{\beta }$), respectively. As shown in Figure 2, this is the top view of the AOTF device and corresponds to the polar plane. In the AOTF, an acoustic wave is generated by a transducer and absorbed by an absorber. By switching the radio frequency signals applied to the transducer, the AOTF can scan the spectral regions of interest [24]. Given that the polarization state of the incident light ($L_{0}$) is inconsistent with one of the eigenwave modes in TeO_2_, four types of emitted light are produced, namely diffraction ordinary polarized light ($L_{d}^{o}$), transmission extraordinary polarized light ($L_{t}^{e}$), transmission ordinary polarized light ($L_{t}^{o}$) and diffraction extraordinary polarized light ($L_{d}^{e}$).

Two coordinate systems have been established for analysis in this paper: the optical axes coordinate system ($x_{0}oy_{0}$) and the crystal axes coordinate system (xoy), as shown in Figure 2. The optical axes coordinate system is a rectangular coordinate system, where the $y_{0}$ axis is the intersection line of the incident surface and polar plane, while the $x_{0}$ axis is perpendicular to the incident surface. The crystal axes coordinate system is also a rectangular coordinate system, wherein the y axis is the crystal axis [110], while the x axis is the crystal axis [001].

**Figure 3:**
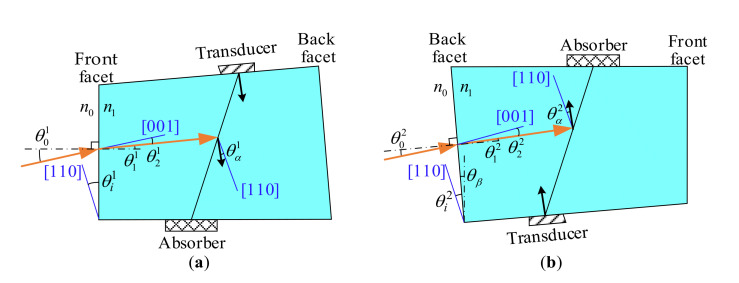
Two modes for analysis. (**a**) Positive mode, lights enter AOTF from the front facet. (**b**) Reverse mode, lights enter AOTF from the back facet.

The model between acousto-optic interaction geometry and polar angular performance for AOTFs involves two processes: (a) the calculation of the refraction at the plane of incidence, shown in Section 2.1, and (b) wave vector analysis of acousto-optic interaction, shown in Section 2.2. In addition, the relationship between the incident polar angles and matching frequencies is independent of the back facet, which will be analyzed in Section 2.1. Therefore, we need two measurements to obtain complete calibration results by swapping the front and back facets, as shown in Figure 3. For the exchange of the input and output facets, the AOTF device needs to be rotated about 180° around the axis perpendicular to the xoy plane.

### 2.1. Refraction at the Plane of Incidence

Firstly, the refraction of light in the plane of incidence obeys Snell’s law as follows:

(1) $$n_{0}\sin\theta_{0}=n_{1}\sin\theta_{1}$$

where $\theta_{0}$ is the incident polar angle between the incident light and the normal of the incident plane. $\theta_{1}$ is the refraction angle in the crystal between the refracted light and the normal to the plane of incidence. $n_{0}$ and $n_{1}$ are the refractive indices in the air and crystal, respectively. Moreover, it is known that $n_{0}$ is equal to 1 in the air. TeO_2_ crystal has the anisotropy and $n_{1}$ can be solved by:

(2) $$\left\{\begin{array}{l} n_{1}^{o}=n^{o}\\ n_{1}^{e}=\frac{n^{o}n^{e}}{\sqrt{{\left(n^{e}\right)}^{2}\cos^{2}\theta_{2}^{e}+{\left(n^{o}\right)}^{2}\sin^{2}\theta_{2}^{e}}} \end{array}\right.$$

with superscripts (*o* and *e*) used to distinguish ordinary (o-polarized) from extraordinary (e-polarized) light. $n^{o}$ and $n^{e}$, related to the wavelength λ, are the principal refractive indices of TeO_2_. $\theta_{2}^{o}$ and $\theta_{2}^{e}$ are the angles between refracted light and the crystal axis [001] for o-polarized and e-polarized lights, respectively. The difference between $\theta_{1}$ and $\theta_{2}$ is as follows:

(3) $$\theta_{2}=\theta_{1}-\theta_{i}^{c}$$

where $\theta_{i}^{c}$ is the angle between the incident plane and the crystal axis [110], for which c=1 corresponds to the positive mode, as shown in Figure 3a, while c=2 corresponds to the reverse mode, as shown in Figure 3b. When switching from the positive mode to the reverse mode, the AOTF device must be rotated for swapping the input and output facets. Furthermore, the relationships between $\theta_{i}^{c}$ and the acousto-optic interaction geometry ($\theta_{i}^{*}$, $\theta_{\alpha }$ and $\theta_{\beta }$) of an AOTF are as follows:

(4) $$\left\{\begin{array}{l} \theta_{i}^{1}=\theta_{i}^{*}\\ \theta_{i}^{2}=\theta_{i}^{*}-\theta_{\beta } \end{array}\right.$$

which means that both the positive and reverse modes are necessary to obtain the complete acousto-optic interaction geometry of an AOTF device. From Equations (1)–(3), we can obtain:

(5) $$\left\{\begin{array}{l} F^{e}(x)=\left(a_{1}a_{3}-a_{2}a_{3}a_{5}^{2}\right)x^{2}-2a_{2}a_{3}a_{4}a_{5}x+a_{1}a_{2}-a_{2}a_{3}a_{4}^{2}=0\\ F^{o}(x)=\left(a_{1}-a_{3}a_{5}^{2}\right)x^{2}-2a_{3}a_{4}a_{5}x+a_{1}-a_{3}a_{4}^{2}=0 \end{array}\right.$$

where $F^{e}$ and $F^{o}$ correspond to the conditions under which the incident lights are the e-polarized and o-polarized, respectively. Furthermore, $a_{1}=\sin^{2}\theta_{0}$, $a_{2}={\left(n^{e}\right)}^{2}$, $a_{3}={\left(n^{o}\right)}^{2}$, $a_{4}=\sin\theta_{i}^{c}$, $a_{5}=\cos\theta_{i}^{c}$ and $x=\tan\theta_{2}$. Equation (5) contains both the quadratic equations and can easily be solved. Then, using Equations (1)–(5), the relationship between $\theta_{0}$ and $\theta_{2}$ can be expressed as follows:

(6) $$\theta_{2}=F_{1}\left(\theta_{0},\lambda ,\theta_{i}^{*},\theta_{\beta }\right)$$

where $F_{1}$ is an implicit function.

### 2.2. Wave Vector Analysis of Acousto-Optic Interaction

**Figure 4:**
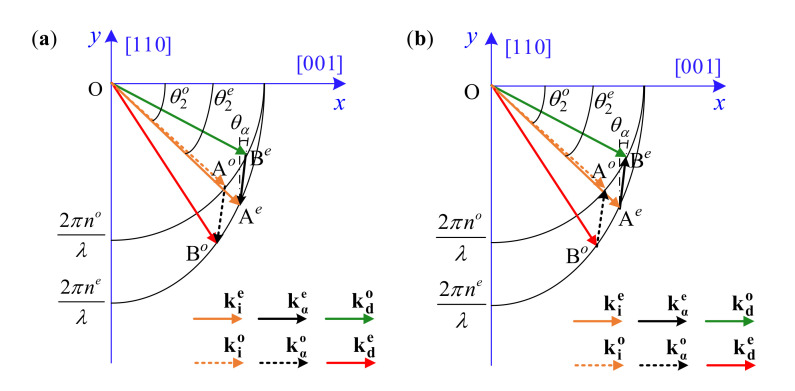
Wave vector diagrams of the noncollinear AOTF. (**a**) Positive mode. (**b**) Reserve mode.

Acousto-optic interaction in the AOTF is usually analyzed through a wave vector diagram [25]. When the momentum-matching condition is satisfied, the incident wave vector $k_{i}$, the acoustic wave vector $k_{\alpha }$ and the diffraction wave vector $k_{d}$ constitute a closed triangle (Figure 4), shown as $k_{i}\pm k_{\alpha }=k_{d}$. Some are dependent on the incident wavelength and refraction indices, as follows:

(7) $$\left\{\begin{array}{l} \left|k_{i}\right|=\frac{2\pi n_{i}}{\lambda }\\ \left|k_{d}\right|=\frac{2\pi n_{d}}{\lambda } \end{array}\right.$$

where $n_{i}$ and $n_{d}$ are the refractive indices of the incident and diffracted light in TeO_2_. They satisfy:

(8) $$\left\{\begin{array}{l} \frac{x^{2}}{{\left(n^{o}\right)}^{2}}+\frac{y^{2}}{{\left(n^{o}\right)}^{2}}={\left(\frac{2\pi }{\lambda }\right)}^{2}\\ \frac{x^{2}}{{\left(n^{o}\right)}^{2}}+\frac{y^{2}}{{\left(n^{e}\right)}^{2}}={\left(\frac{2\pi }{\lambda }\right)}^{2} \end{array}\right.$$

In addition, acoustic wave vector $k_{\alpha }$ satisfies:

(9) $$\left|k_{a}\right|=\left|\vec{\mathrm{AB}}\right|=\frac{2\pi f_{\alpha }}{V_{\alpha }}$$

where $f_{\alpha }$ is the acoustic frequency and $V_{\alpha }$ is the acoustic wave velocity. In the crystal, $V_{\alpha }$ is given by [26]:

(10) $$V_{\alpha }^{2}=V_{110}^{2}\cos^{2}\theta_{\alpha }+V_{001}^{2}\sin^{2}\theta_{\alpha }$$

where $V_{001}$ and $V_{110}$ are the acoustic wave velocities along the respective crystal axes. According to Equation (9), in order to solve acoustic frequency $f_{\alpha }$, we need to calculate the $\left|\vec{\mathrm{AB}}\right|$. As shown in Figure 4, point A $\left(x_{A},y_{A}\right)$satisfies tanθ2=yAxA and Equation (8), which can be solved by following:

(11) $$\left\{\begin{array}{l} A^{o}=\left(1,\tan\theta_{2}^{o}\right)\frac{2\pi n^{o}}{\lambda \cdot \sqrt{1+\tan^{2}\theta_{2}^{o}}}\\ A^{e}=\left(1,\tan\theta_{2}^{e}\right)\frac{2\pi n^{o}n^{e}}{\lambda \cdot \sqrt{{\left(n^{e}\right)}^{2}+{\left(n^{o}\right)}^{2}\tan^{2}\theta_{2}^{e}}} \end{array}\right.$$

Point B $\left(x_{B},y_{B}\right)$ satisfies the linear equation of $\vec{\mathrm{AB}}$ and Equation (8). For both positive and reverse modes, the linear equations of $\vec{\mathrm{AB}}$ are the same one as:

(12) $$\frac{x_{B}-x_{A}}{y_{B}-y_{A}}=\tan\theta_{\alpha }$$

Therefore, point B can be solved by following:

(13) $$\left\{\begin{array}{cc} \; & F^{o\to e}\left(y_{B}\right)=\left(\frac{\tan^{2}\theta_{\alpha }}{{\left(n^{o}\right)}^{2}}+\frac{1}{{\left(n^{e}\right)}^{2}}\right){y_{B}}^{2}+\frac{2}{{\left(n^{o}\right)}^{2}}\left(x_{A}\tan\theta_{\alpha }-y_{A}\tan^{2}\theta_{\alpha }\right)y_{B}\\ \; & \;+\left(\frac{{x_{A}}^{2}+{y_{A}}^{2}\tan^{2}\theta_{\alpha }-2x_{A}y_{A}\tan\theta_{\alpha }}{{\left(n^{o}\right)}^{2}}-\frac{2\pi }{\lambda }\right)=0\\ \; & F^{e\to o}\left(y_{B}\right)=\left(\frac{\tan^{2}\theta_{\alpha }}{{\left(n^{o}\right)}^{2}}+\frac{1}{{\left(n^{o}\right)}^{2}}\right){y_{B}}^{2}+\frac{2}{{\left(n^{o}\right)}^{2}}\left(x_{A}\tan\theta_{\alpha }-y_{A}\tan^{2}\theta_{\alpha }\right)y_{B}\\ \; & \;+\left(\frac{{x_{A}}^{2}+{y_{A}}^{2}\tan^{2}\theta_{\alpha }-2x_{A}y_{A}\tan\theta_{\alpha }}{{\left(n^{o}\right)}^{2}}-\frac{2\pi }{\lambda }\right)=0 \end{array}\right.$$

which are all quadratic equations and easy to be solved exactly. $F^{o\to e}$ corresponds to the condition wherein the incident light is o-polarized and the diffraction light is e-polarized, while $F^{e\to o}$ corresponds to the condition wherein the incident light is e-polarized and the diffraction light is o-polarized. From Equations (9)–(13), the relationship between $f_{\alpha }$ and $\theta_{2}$ can be expressed as follows:

(14) $$f_{\alpha }=F_{2}\left(\theta_{2},\lambda ,\theta_{\alpha }\right)$$

where $F_{2}$ is an implicit function.

In summary, with Equations (6) and (14), the model between the acousto-optic interaction geometry and the incident polar angular frequencies of AOTFs can be established as follows:

(15) $$f_{\alpha }=F\left(\theta_{0},\lambda ,\theta_{i}^{*},\theta_{\alpha },\theta_{\beta }\right)$$

where F is an implicit function. This means that, for an actual AOTF device with inherent acousto-optic interaction geometry ($\theta_{i}^{*}$, $\theta_{\alpha }$ and $\theta_{\beta }$), acoustic frequencies and incident polar angles are correlated when the wavelength (λ) of the incident lights is fixed. Therefore, the acousto-optic interaction geometry of the AOTF device can be calibrated by analyzing the incident polar angles and corresponding acoustic frequencies.

The numerical values of the constants used in the calculations in this paper are provided in Table 1 [27].

## 3. Experiments and Discussions

### 3.1. Experimental Setup

**Figure 5:**
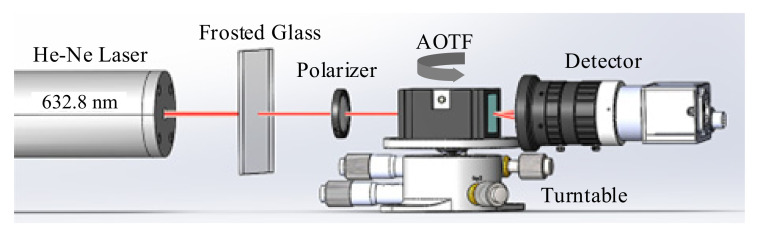
Experimental setup for the polar angular frequency tests.

**Figure 6:**
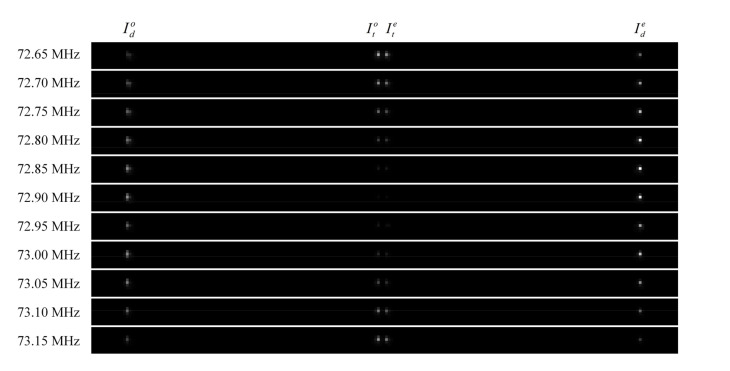
A montage of 11 images taken at different frequencies in sequence. The incident polar angle is 2.19° in the positive mode.

The schematic diagram of our experimental setup is shown in Figure 5. The monochromatic source we used was a 632.8 nm He-Ne laser (DH-HN250), from which the linearly polarized light was generated. A group of frosted glasses was used to reduce light intensity, and the effect of the frosted glasses can be replaced by multiple polarizers. The commercial AOTF used in the experiment was manufactured by China Electronics Technology Group Corporation (CETC) and is referred to as SGL100-400/850-20LG-K. The polarizer, located ahead of the AOTF, was used to adjust the polarization state of incident lights so that o-polarized and e-polarized components were close and convenient for measurements. The turntable (GCM-1107M) was used to accurately control the incident polar angles of the incident light into the AOTF, and here the accuracy of the rotation angle was 2′. A Basler acA640-120gm camera with an 8 mm focal length lens was used as the detector in the experiments. Compared with the optical power meter, a camera can simultaneously detect the intensities of transmitted and diffracted lights (Figure 6) to effectively avoid the measurement error caused by the instability of the power intensity and the polarization state of the laser.

### 3.2. Results and Discussions

**Figure 7:**
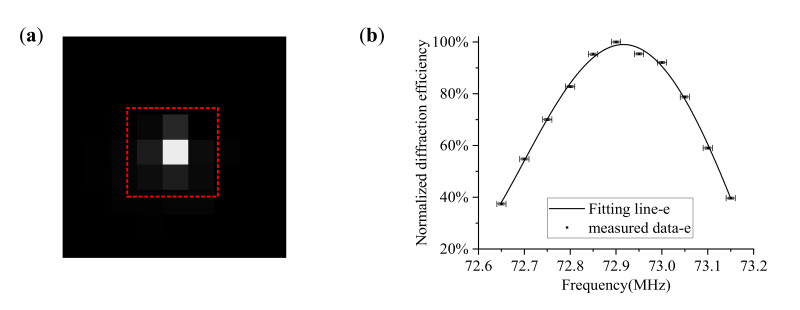
Experimental data for incident polar angular frequency analysis. (**a**) The effective DN value is the sum of the DN values within 9 adjacent pixels, and the 9 adjacent pixels are here surrounded by red dotted lines here; (**b**) The data fitting result by quartic polynomial fitting, and the measured data can be found in Figure 6.

For incident polar angle analysis, we needed to measure the matching frequency at each incident polar angle, which corresponds to the peak diffraction intensity. In practice, the potential range of the matching frequency can be estimated from Equation (15) using the design geometry parameters from the AOTF manufacturer or through the direct observation of the maximum diffraction intensity. It should be noted that in some special applications, the changing ultrasonic signal and the angle of the incident light will greatly change the shape of the AOTF transfer function, which needs further discussion [28,29]. The processes of the tests are organized as follows:

- Step 1: Adjust the polar angle of the AOTF by using the turntable and make sure that the incident plane of the AOTF is perpendicular to the incident light. This step can be judged by whether the reflected laser point coincides with the exit point. We recorded the scale of the turntable at this point as the “0” scale, and the other incident polar angles were able to be adjusted with this scale.
- Step 2: After adjusting the AOTF incident polar angle, the laser, AOTF and detector must be switched on. Then a montage of images, including transmitted and diffracted light, can be taken by scanning the acoustic frequencies, as shown in Figure 6. For each image, both transmitted and diffracted light can be captured, or only o-polarized and e-polarized light can be measured separately by adjusting the polarizer. Given that, in some cases, the AOTFs do not have the wedge angle compensation, the directions of transmitted o-polarized and e-polarized light are coincident. In these experiments, the frequency step was 0.05 MHz.
- Step 3: To find the matching frequency corresponding to the peak diffraction intensity, use the relative diffraction efficiency to evaluate as:
  (16) η=IdId+Itηmax
  where $I_{t}$ and $I_{d}$ are the light intensity values for the transmitted and diffracted light from the same incident light. The intensity values are quantified by the digital number (DN) values with 8-bit digitization. For each order of emitted light, we used the sum of DN values in the effective area, where nine adjacent pixels were selected for calculation, as shown in Figure 7a.
- Step 4: The matching frequencies were able to be solved by quartic polynomial fitting, as shown in Figure 7b, and at least five frequency points are required for each incident polar angle.
- Step 5: In order to ensure that the temperature of each measurement is close to the room temperature, the AOTF needs to be switched off for a few minutes because the temperature of the AOTF rises during operation, which would affect its polar angular performance.
- Step 6: Adjust another incident polar angle of the AOTF, switch on the AOTF and repeat Steps 2–6 again.

**Figure 8:**
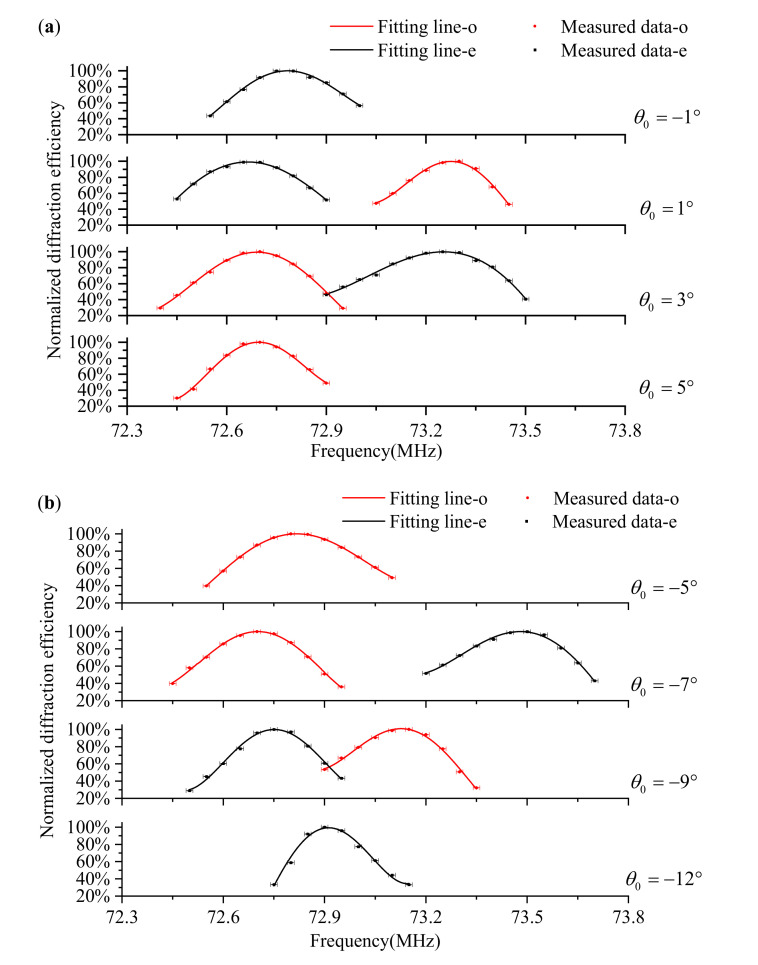
Some other data fitting results. (**a**) Positive mode, normalized diffraction efficiencies by frequency scanning at some incident polar angles (−1°, 1°, 3°, 5°); (**b**) reverse mode, normalized diffraction efficiencies by frequency scanning at some incident polar angles (−5°, −7°, −9°, −12°).

The coefficients of determination ($R^{2}$) of all the results were better than 0.98, and the fitting residuals were less than 0.003. Some other data fitting results at different incident polar angles are shown in Figure 8. From these results, we found that the matching frequencies of o-polarized and e-polarized lights are generally not consistent under the same incident polar angles. In other words, at the same acoustic frequency, the diffracted wavelengths of the o-polarized and e-polarized lights are not consistent. However, under a specific incident polar angle, we obtained the same diffracted wavelengths of the o-polarized and e-polarized lights at the same acoustic frequency. In some research, they named this condition the equivalent point, wherein the matching frequencies are the same for o-polarized and e-polarized light at the same incident polar angle [30,31]. Here, we obtained this condition by adjusting the incident polar angle. Moreover, it would be exactly calculated with an exact acousto-optic interaction geometry of the AOTF. Therefore, we will discuss it after the acousto-optic interaction geometry calibration.

**Figure 9:**
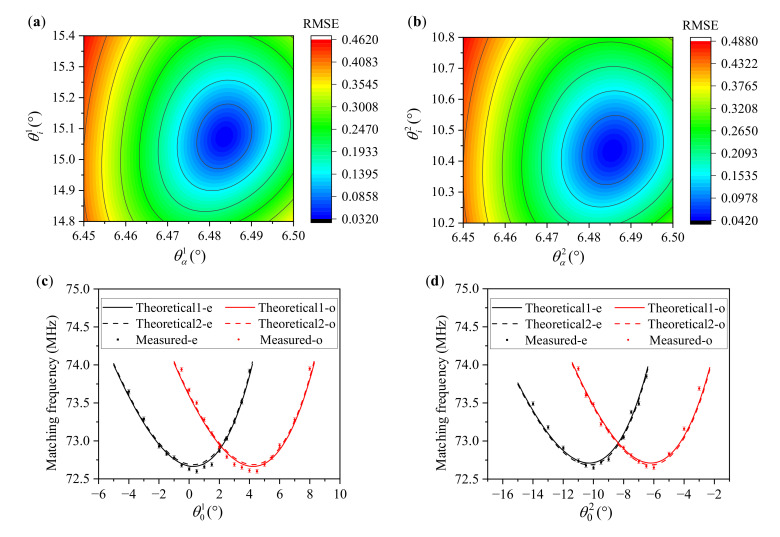
Calibration results of the actual AOTF device. (**a**) RMSE distribution in the positive mode; (**b**) RMSE distribution in the reserve mode; (**c**) measured data and theoretical simulation curves in the positive mode. The solid line marked “Theoretical-1e” is the theoretical simulation curve under which $\theta_{i}^{1}=15.074{}^{\circ}$ and $\theta_{\alpha }^{1}=6.484{}^{\circ}$, while the dashed line marked “Theoretical-2e” is the theoretical simulation curve under which $\theta_{i}^{1}=15.074{}^{\circ}$ and $\theta_{\alpha }^{1}=6.485{}^{\circ}$; (**d**) measured data and theoretical simulation curves in the reserve mode. The solid line marked “Theoretical-1e” is the theoretical simulation curve under which $\theta_{i}^{2}=10.435{}^{\circ}$ and $\theta_{\alpha }^{2}=6.486{}^{\circ}$, while the dashed line marked “Theoretical-2e” is the theoretical simulation curve under which $\theta_{i}^{2}=10.435{}^{\circ}$ and $\theta_{\alpha }^{2}=6.485{}^{\circ}$.

In this paper, a total of 21 incident polar angles were sampled in the positive mode, while 17 incident polar angles were sampled in the reverse mode. In the experiments, the minimum angle sampling step was 0.5°. All of the matching frequencies in positive and reserve modes can be found in Figure 9c,d. After sampling all of the measured data, we used the parameter traversal method to calculate the acousto-optic interaction geometry of the AOTF with the principle of minimum RMSE. For each input of the geometry parameters, the RMSE between the theoretical data and measured data is as follows:

(17) $$RMSE=\sqrt{\frac{1}{N}{\sum_{i=1}^{N}\left|F\left(\theta_{0}(i),\lambda_{0},\theta_{i}^{*},\theta_{\alpha },\theta_{\beta }\right)-F_{m}\left(\theta_{0}(i)\right)\right|}^{2}}$$

where N is the number of sampling points for each mode, and there are two types of sampling points for both o-polarized and e-polarized lights at some incident polar angles. N was 31 for the positive mode in this paper, and 26 for the reserve mode. The $\theta_{0}(i)$ is the incident polar angle, and the incident wavelength ($\lambda_{0}$) was 632.8 nm. $F_{m}$ is the measured data of the matching frequency at each incident polar angle. As shown in Figure 9, we obtained the RMSE distributions in both positive and reserve modes. Then, the acousto-optic interaction geometry of the AOTF device, corresponding to the minimum RMSE, was able to be obtained. As shown in Figure 9a, we obtained $\theta_{i}^{1}=15.074{}^{\circ}$ and $\theta_{\alpha }^{1}=6.484{}^{\circ}$ in the positive mode with the minimum RMSE (0.032 MHz). Meanwhile, we also obtained $\theta_{i}^{2}=10.435{}^{\circ}$ and $\theta_{\alpha }^{2}=6.486{}^{\circ}$ in the reserve mode with the minimum RMSE (0.042 MHz) in Figure 9b. The difference of $\theta_{\alpha }$ between the two measurements was 0.002°, which means this calibration method has a high precision and can be better than 0.01°. We took the average of two results as the calibration value that $\theta_{\alpha }=6.485{}^{\circ}$. As shown in Figure 9c,d, the measured data were very close to the theoretical data. In summary, we obtained the calibration results of $\theta_{i}^{*}=15.074{}^{\circ}$, $\theta_{\alpha }=6.485{}^{\circ}$ and $\theta_{\beta }=4.639{}^{\circ}$ with Equation (4). In addition, according to the reference [19], we calculated the acousto-optic interaction geometry, meeting the parallel tangent condition, whereby $\theta_{i}^{*}=15.074{}^{\circ}$ and $\theta_{\alpha }=6.548{}^{\circ}$ under normal incidence of e-polarized light at 632.8 nm. Therefore, we found that the calibration result of the actual AOTF device was close but did not meet the parallel tangent condition at 632.8 nm.

**Figure 10:**
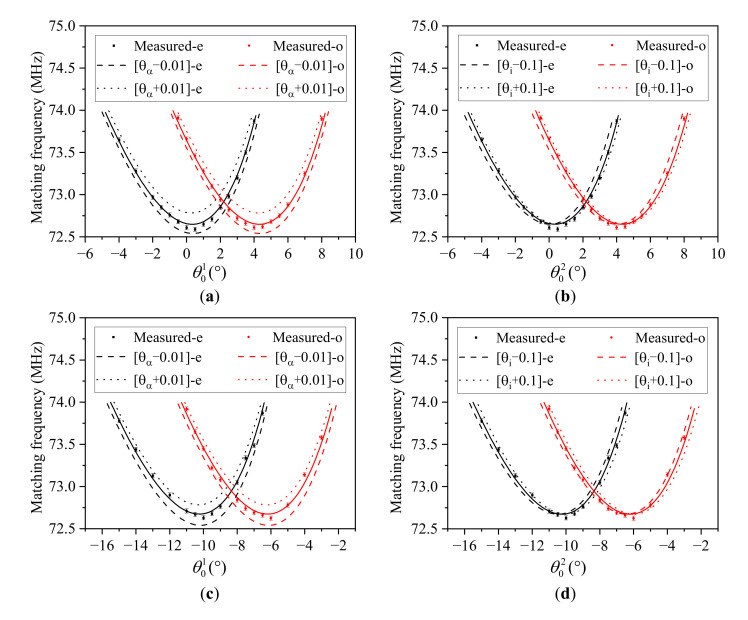
Polar angular frequency analysis under the different acoustic cut angles and front facet angles. (**a**,**b**) are both in the positive mode, while (**c**,**d**) are both in the reserve mode. The solid lines in (**a**,**c**) are the theoretical simulation curves under the calibration result whereby $\theta_{i}^{1}=15.074{}^{\circ}$ and $\theta_{\alpha }^{1}=6.485{}^{\circ}$; while the solid lines in (**b**,**d**) are the theoretical simulation curves under the calibration result whereby $\theta_{i}^{2}=10.435{}^{\circ}$ and $\theta_{\alpha }^{2}=6.485{}^{\circ}$. The dashed lines in (**a**,**c**) marked “$\left[\theta_{\alpha }-0.01{}^{\circ}\right]$” are the theoretical simulation curves under which $\theta_{\alpha }=6.475{}^{\circ}$, while the dotted lines in (**a**,**c**) marked “$\left[\theta_{\alpha }+0.01{}^{\circ}\right]$” are the theoretical simulation curves under which $\theta_{\alpha }=6.495{}^{\circ}$. The dashed lines in (**b**) and (**d**) marked “$\left[\theta_{i}-0.1{}^{\circ}\right]$” are the theoretical simulation curves under which $\theta_{i}^{1}=14.974{}^{\circ}$ and $\theta_{i}^{2}=10.335{}^{\circ}$, respectively, while the dotted lines in (**b**,**d**) marked “$\left[\theta_{i}+0.1{}^{\circ}\right]$” are the theoretical simulation curves under which $\theta_{i}^{1}=15.174{}^{\circ}$ and $\theta_{i}^{2}=10.535{}^{\circ}$, respectively.

From Figure 9a,b, we found that the acoustic cut angle of the AOTF was more sensitive than the front facet angle. Therefore, we further analyzed the angular frequency relationship under the different acoustic cut angles and front facet angles, and the results are shown in Figure 10. The deviation caused by the change of acoustic cut angles (±0.01°) was higher than that caused by the change of front facet angles (±0.1°). These results confirm that changing the acoustic cut angle has a greater influence. Moreover, changing the acoustic cut angle makes the angular frequency curves shift up and down, and the larger acoustic cut angle corresponds to the state of shifting up. In comparison, changing the front facet angle makes the angular frequency curves shift left and right, and the larger front facet angle corresponds to the state of shifting right.

**Figure 11:**
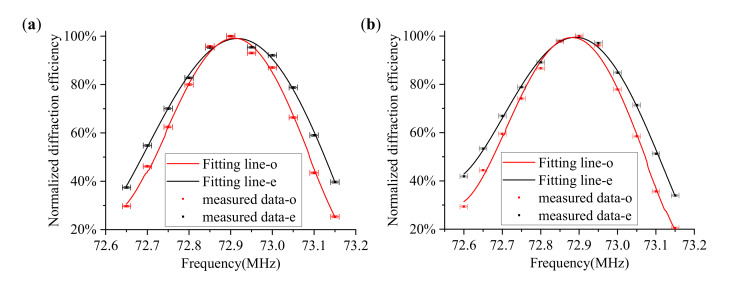
Measurement results at the equivalent points. (**a**) Incident polar angle at 2.19° in the positive mode. (**b**) Incident polar angle at −8.34° in the reserve mode.

In order to further verify the accuracy of the calibration result, the equivalent points in two modes are calculated and tested here. According to the calibration results and Equation (15), the equivalent points can be solved as the incident polar angle is 2.19° in the positive mode and −8.34° in the reserve mode. The measured results are shown in Figure 11a,b, respectively. The results show that the matching frequencies are approximately the same at the same incident polar angle when ignoring the bandwidth for o-polarized and e-polarized lights. Furthermore, the differences in the peak diffraction efficiency are both less than 0.01 MHz. This work is significant for non-polarization AOTF applications.

### 3.3. Tolerance Analysis

The constant parameters and measurement parameters involved in the calibration method may have some tolerances, which have not been taken into account above. The constant parameters mainly include the principal refractive index tolerance and the acoustic wave velocity tolerance. The principal refractive index tolerance was taken from reference [27], and the acoustic wave velocity tolerance was set to ±0.5 m/s here. The measurement parameters mainly include the rotational accuracy of the precision turntable (2′) and the sampling step of the tuning frequency (0.05 MHz). The specific range of the tolerance setting can be found in Table 2, and the tolerance distribution of all parameters assumes the uniform distribution probability.

**Figure 12:**
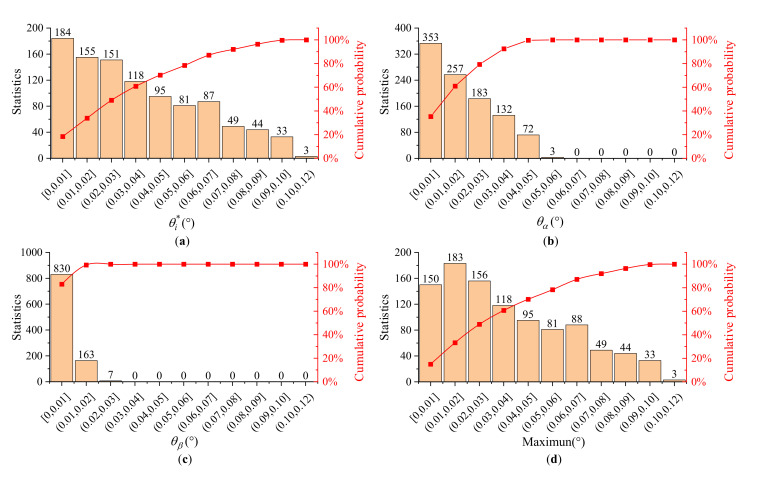
Monte Carlo simulation results. (**a**) Statistical distribution of the front facet angle deviations. (**b**) Statistical distribution of the acoustic cut angle deviations; (**c**) Statistical distribution of the back facet angle deviations. (**d**) Statistical distribution of the maximum cut angle deviations.

We performed a tolerance analysis on the calibration method. The tolerance analysis includes two aspects: parameter sensitivity analysis and Monte Carlo analysis [32]. The variable-controlled method was used to analyze the parameter sensitivity, wherein only one of the parameters varied for 100 times at a time. The standard deviations of the calibration results were used as the parameter sensitivity analysis index. The results show that the principal refractive index tolerance had the greatest influence on the calibration results, especially for the front facet angle and the acoustic cut angle. In comparison, other parameter tolerances had very little effect. Monte Carlo analysis was then used to analyze the statistical tolerance of the entire calibration process. In this paper, a total of 1000 simulated calibrations were performed as the statistical sample in Figure 12. The statistical results of the error distribution of the calibration results are shown in Table 3, and the cumulative probability of a result within than 0.1° was greater than 99.7%. Moreover, the cumulative probability of the front facet angle falling within 0.01° was greater than 18.4%. The cumulative probability of an acoustic cut angle falling within 0.01° was greater than 35.3%. The cumulative probability of a back facet angle falling within 0.01° was greater than 83.0%. Furthermore, the cumulative probability of maximum a cut-angle deviation falling within 0.01° was greater than 15.0%.

## 4. Conclusions

In summary, we proposed a method for calibrating the acousto-optic interaction geometry of AOTFs based on polar angular analysis. Moreover, based on this method, we obtained the complete acousto-optic interaction geometry of an actual AOTF device, including the front facet angle ($\theta_{i}^{*}$), the acoustic cut angle ($\theta_{\alpha }$) and the back facet angle ($\theta_{\beta }$). Specifically, we carried out the following research:

(a) We established a model of the AOTF angular frequency relationship that can be solved analytically.
(b) We proposed a novel and easy-to-perform method for calibrating the acousto-optic interaction geometry of an actual AOTF device. Furthermore, the experimental results showed a high precision with the acoustic cut angle, within results falling within 0.01°.
(c) We analyzed the polar angular performance with the acousto-optic interaction geometry of the AOTF and the results showed that the acoustic cut angle of the AOTF is more sensitive than the front facet angle. Specifically speaking, changing the acoustic cut angle makes the angular frequency curves shift up and down, and the larger acoustic cut angle corresponds to the state of shifting up. In comparison, changing the front facet angle makes the angular frequency curves shift left and right, and the larger front facet angle corresponds to the state of shifting right.
(d) We calculated and tested the equivalent points for the o-polarized and e-polarized lights in both positive and reserve modes, which is vital to the non-polarization applications of AOTFs.
(e) We analyzed the parameter sensitivity and Monte Carlo tolerance of the calibration method. The results of the parameter sensitivity analysis showed that the principal refractive index of the crystal has a large influence on the calibration results, while other factors have little influence. The results of the Monte Carlo tolerance analysis showed that the cumulative probability of the results falling within 0.1° with this method is greater than 99.7%. Moreover, the probability of falling within 0.01°, for the front facet angle is greater than 18.4%. In comparison, the acoustic cut angle and the back facet angle are greater than 35.3% and 83.0%, respectively.

These works are of great significance for the studies of AOTFs, such as ray tracing, characteristic analysis and optical system design.

## Figures and Tables

**Table 1:** The numerical value of the constants used in the calculations.

Parameters	Values
λ	632.8 nm
$n^{o}$	2.2597 at 632.8 nm
$n^{e}$	2.4119 at 632.8 nm
$V_{001}$	616 m/s
$V_{110}$	2104 m/s

**Table 2:** Tolerance setting and standard deviation of calibration results by the variable-controlled method.

Parameters		Tolerances	Standard Deviation of Calibration Results		
			$\theta_{i}^{*}$	$\theta_{\alpha }$	$\theta_{\beta }$
Principle refractive index	$n^{o}$	±0.0006	0.066°	0.032°	0.001°
	$n^{e}$	±0.0007			
Acoustic wave velocity	$V_{001}$	±0.5 m/s	0.002°	0.001°	0.001°
	$V_{110}$				
Incident polar angle		±1′	0.003°	<0.001°	0.006°
Matching frequency		±0.025 MHz	0.004°	<0.001°	0.005°

**Table 3:** Cumulative probability of the Monte Carlo analysis result.

Parameters	≤0.01°	≤0.1°
$\theta_{i}^{*}$	18.4%	99.7%
$\theta_{\alpha }$	35.3%	100%
$\theta_{\beta }$	83.0%	100%
Maximum	15.0%	99.7%

## Data Availability

Not applicable.
